# Supplementary material for: Adipocyte-derived kynurenine promotes obesity and insulin resistance by activating the AhR/STAT3/IL-6 signaling
Source: Nat Commun. 2022 Jun 17;13:3489. doi: 10.1038/s41467-022-31126-5 (PMC9205899; doi:10.1038/s41467-022-31126-5)
Supplement: Supplementary file 1 — Supplementary Information [file 41467_2022_31126_MOESM1_ESM.pdf]

Supplementary Materials for

Adipocyte-Derived Kynurenine Promotes Obesity and Insulin Resistance by Activating the AhR/STAT3/IL-6 Signaling

Teng Huang[1,6], Jia Song[2,6], Jia Gao[1,6], Jia Cheng[3,6], Hao Xie[1], Lu Zhang[1], Yuhan Wang[1], Zhichao Gao[1], Yi Wang[1], Xiaohui Wang[1], Jinhan He[4], Shiwei Liu[5], Qilin Yu[1], Shu Zhang[1], Fei Xiong[1*], Qing Zhou[1*], and Cong-Yi Wang[1*].

[1]Department of Respiratory and Critical Care Medicine, the Center for Biomedical Research, NHC Key Laboratory of Respiratory Diseases, Tongji Hospital, Tongji Medical College, Huazhong University of Science and Technology, Wuhan, China;

[2]Reproductive Medicine Center, Tongji Hospital, Tongji Medical College, Huazhong University of Science and Technology, Wuhan, China;

[3]Division of Cardiology, Department of Internal Medicine, Tongji Hospital, Tongji Medical College, Huazhong University of Science and Technology, Wuhan, China;

[4]Department of Pharmacy, National Clinical Research Center for Geriatrics, West China Hospital, Sichuan University, Chengdu, China;

[5]Shanxi Bethune Hospital, Shanxi Academy of Medical Sciences, Tongji Shanxi Hospital, Third Hospital of Shanxi Medical University;

[6]These authors contributed equally: Teng Huang, Jia Song, Jia Gao and Jia Cheng;

[*]e-mail: feixiong@tjh.tjmu.edu.cn; zhouqing@tjh.tjmu.edu.cn; wangcy@tjh.tjmu.edu.cn

This PDF file includes:

Supplementary Figures 1 to 10

Supplementary Tables 1 to 3

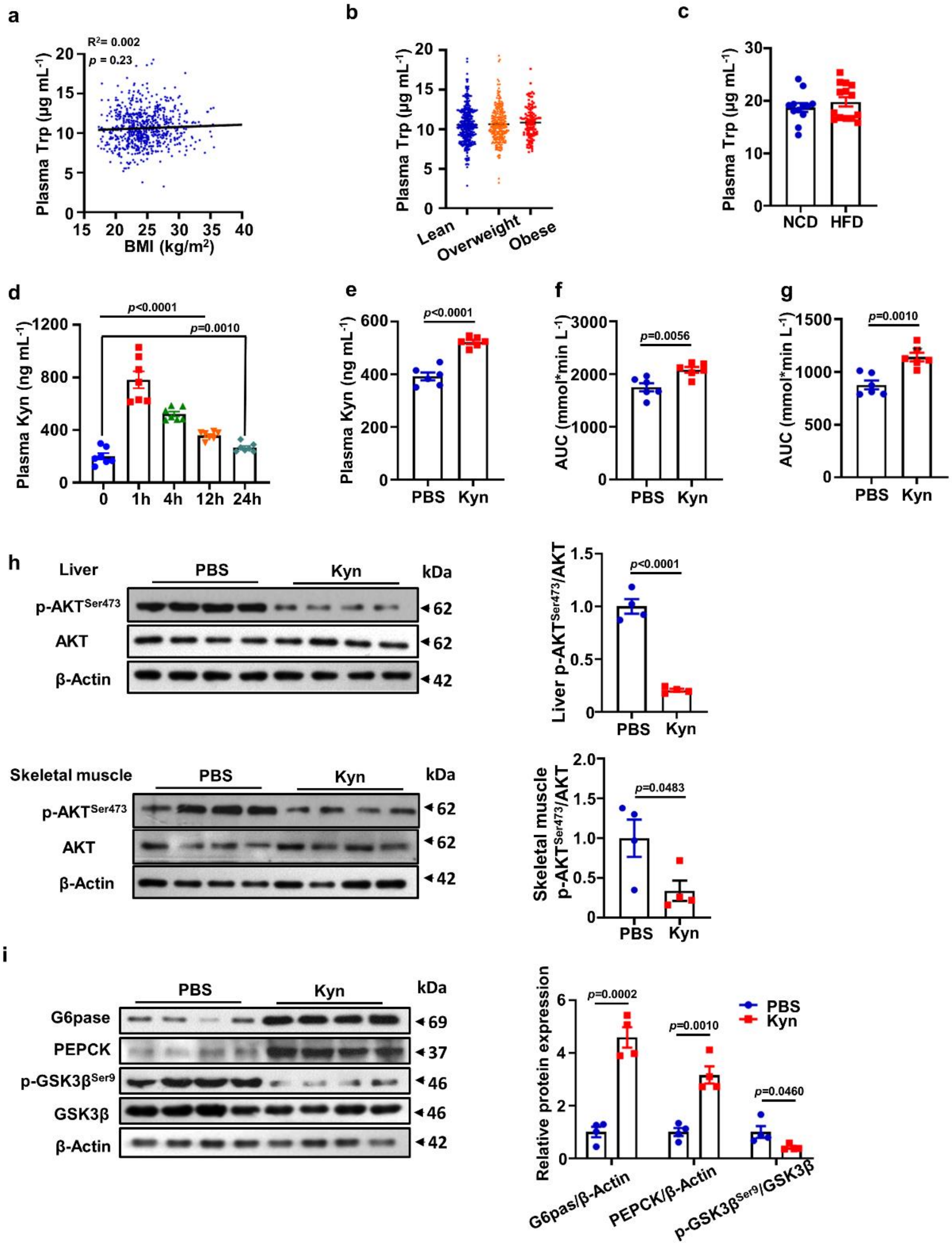

Supplementary Figure 1. Kyn exacerbates insulin resistance in subjects with obesity. a, Correlation analysis between BMI and plasma Trp levels in human samples (n = 735). **b**, Plasma Trp levels in lean subjects (BMI < 24, n = 354), subjects with overweight (24 ≤ BMI < 28, n = 268) and subjects with obesity (BMI ≥ 28, n = 113). **c**, Plasma Trp levels of WT mice fed with

NCD (*n* = 12) or HFD (*n* = 16). **d**, Plasma concentration of Kyn at 0h, 1h, 4h, 12h and 24h post injection (*n* = 6). **e**, Plasma Kyn levels in PBS-treated and Kyn-treated HFD mice (*n* = 6). **f**, **g**, GTT (**f**) and ITT (**g**) in Kyn-treated and PBS-treated HFD mice were respectively calculated by AUC (*n* = 6). **h**, Western blot analysis of p-AKT^{Ser473} and AKT in liver and skeletal muscle from PBS-treated and Kyn-treated mice (*n* = 4). **i**, Western blot of G6pase, PEPCK and p-GSK3β^{Ser9} in liver of PBS-treated and Kyn-treated mice. (*n* = 4). Data were represented as mean ± SEM. Statistical significance was assessed by two-sided Spearman's correlation (**a** and **d**), one-way ANOVA (**b** and **d**), two-way ANOVA (**d**) or two-sided Student's *t*-test (**c**, **e-h**) and significant differences were indicated with *p* values. Source data are provided in the Source Data file.

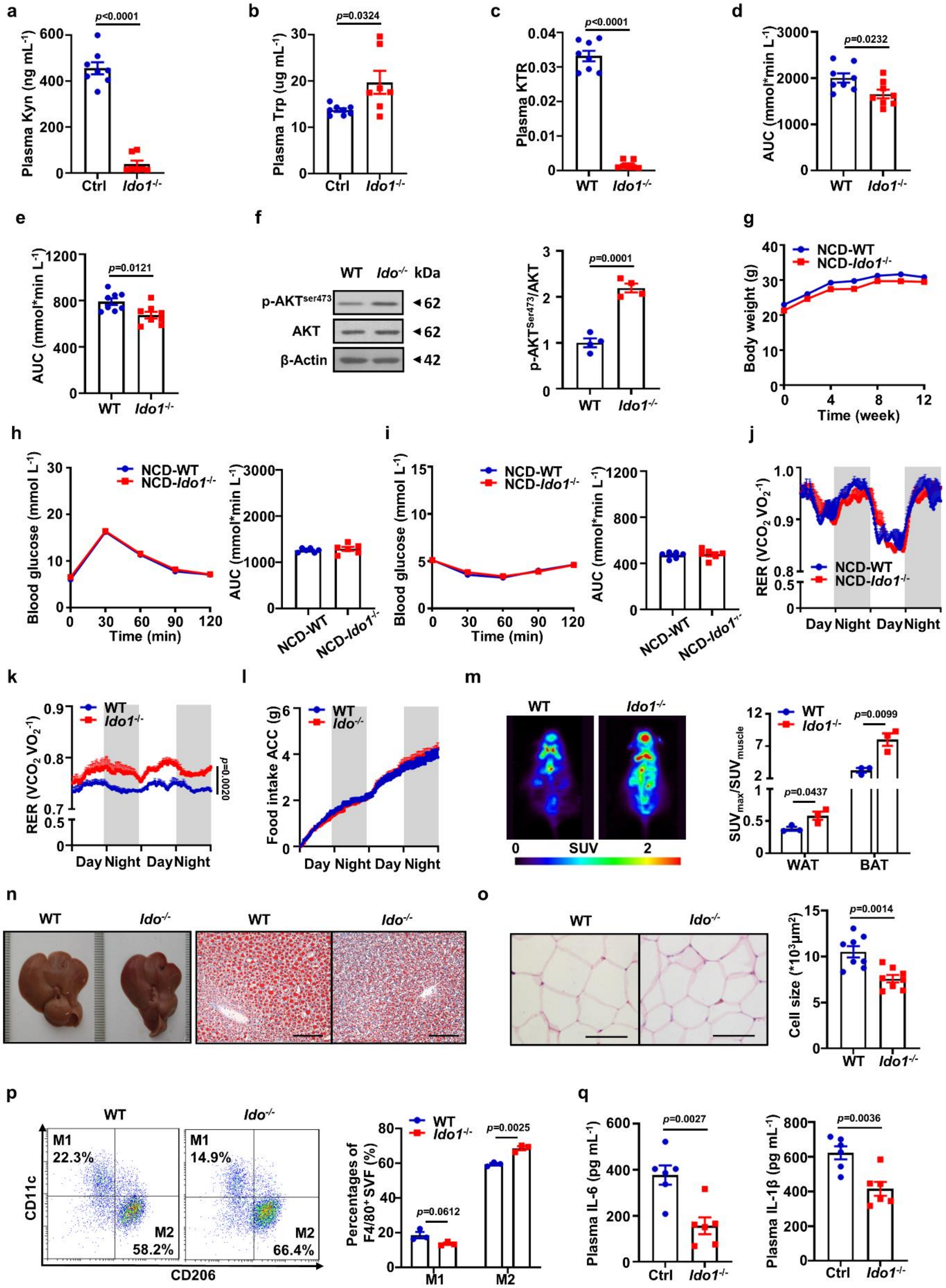

a
p<0.0001
Plasma Kyn (ng mL-1)
Ctrl
Ido1-/-
b
p=0.0324
Plasma Trp (ug mL-1)
Ctrl
Ido1-/-
c
p<0.0001
Plasma KTR
WT
Ido1-/-
d
p=0.0232
AUC (mmol*min L-1)
WT
Ido1-/-
e
p=0.0121
AUC (mmol*min L-1)
WT
Ido1-/-
f
WT
Ido-/-
kDa
p-AKTser473
AKT
β-Actin
62
62
42
p=0.0001
p-AKTSer473/AKT
WT
Ido1-/-
g
NCD-WT
NCD-Ido1-/-
Body weight (g)
Time (week)
h
NCD-WT
NCD-Ido1-/-
Blood glucose (mmol L-1)
Time (min)
AUC (mmol*min L-1)
NCD-WT
NCD-Ido1-/-
i
NCD-WT
NCD-Ido1-/-
Blood glucose (mmol L-1)
Time (min)
AUC (mmol*min L-1)
NCD-WT
NCD-Ido1-/-
j
RER (VCO2 VO2-1)
NCD-WT
NCD-Ido1-/-
Day Night Day Night
k
WT
Ido1-/-
RER (VCO2 VO2-1)
p=0.0020
Day Night Day Night
l
WT
Ido-/-
Food intake ACC (g)
Day Night Day Night
m
WT
Ido1-/-
0
SUV
2
WT
Ido1-/-
SUVmax/SUVmuscle
p=0.0099
p=0.0437
WAT
BAT
n
WT
Ido-/-
WT
Ido-/-
o
WT
Ido-/-
p=0.0014
Cell size (*10³μm²)
WT
Ido1-/-
p
WT
Ido-/-
M1
22.3%
M2
58.2%
M1
14.9%
M2
66.4%
CD11c
CD206
WT
Ido1-/-
p=0.0025
p=0.0612
Percentages of F4/80+ SVF (%)
M1
M2
q
p=0.0027
Plasma IL-6 (pg mL-1)
Ctrl
Ido1-/-
p=0.0036
Plasma IL-1β (pg mL-1)
Ctrl
Ido1-/-

Supplementary Figure 2. IDO1-catalyzed Kyn exacerbates insulin resistance in subjects with obesity. **a**, **b**, **c**, Plasma Kyn levels (**a**), Trp levels (**b**) and KTR (**c**) of WT (*n* = 8) and *Ido*$^{-/-}$ mice (*n* = 7) with 12-week HFD. **d**, **e**, GTT (**d**) and ITT (**e**) of WT and *Ido*$^{-/-}$ mice with 12-week HFD were respectively calculated by AUC (*n* = 8). **f**, Western blot analysis of p-AKT^{Ser473} and AKT in eWAT from WT and *Ido*$^{-/-}$ mice with 12-week HFD (*n* = 4). **g,** Body weights of WT and *Ido*$^{-/-}$ mice fed with NCD for 12 weeks (*n* = 8). **h**, **i**, GTT (**h**) and ITT (**i**) in WT and *Ido*$^{-/-}$ mice fed with NCD for 12 weeks (*n* = 8). **j**, RER was determined by metabolic cages in WT and *Ido*$^{-/-}$ mice with 12 weeks NCD (*n* = 3). **k**, **l**, RER (**k**) and food consumption (**l**) were determined by metabolic cages in WT and *Ido*$^{-/-}$ mice with 12 weeks HFD (*n* = 4). **m**, Representative images of 18F-FDG uptake (left) and relative quantification (right) in WT and *Ido*$^{-/-}$ mice fed with HFD for 12 weeks (*n* = 3). **n**, Representative images of livers dissected from 12-week HFD fed WT and *Ido*$^{-/-}$ mice (left) and the Oil Red staining (right, *n* = 6). The experiments were repeated independently three times. Scale bar, 100 µm. **o**, Representative H&E staining images of eWAT from 12-week HFD WT and *Ido*$^{-/-}$ mice (left) and the calculation of adipocyte size (right) (*n* = 8). Scale bar, 100 µm. **p**, Flow cytometry analysis of macrophage subsets in eWAT of WT and *Ido*$^{-/-}$ mice fed with 12-week HFD (*n* = 8). **q**, Plasma IL-6 and IL-1β levels of WT and *Ido*$^{-/-}$ mice with 12-week HFD were detected by ELISA (*n* = 6). Data were represented as mean ± SEM. Statistical significance was assessed by two-sided Student's *t*-test (**a-f**, **m**, **o-q**) or two-way ANOVA followed with Bonferroni's multiple comparisons test (**g-l**) and significant differences were indicated with *p* values. Source data are provided in the Source Data file.

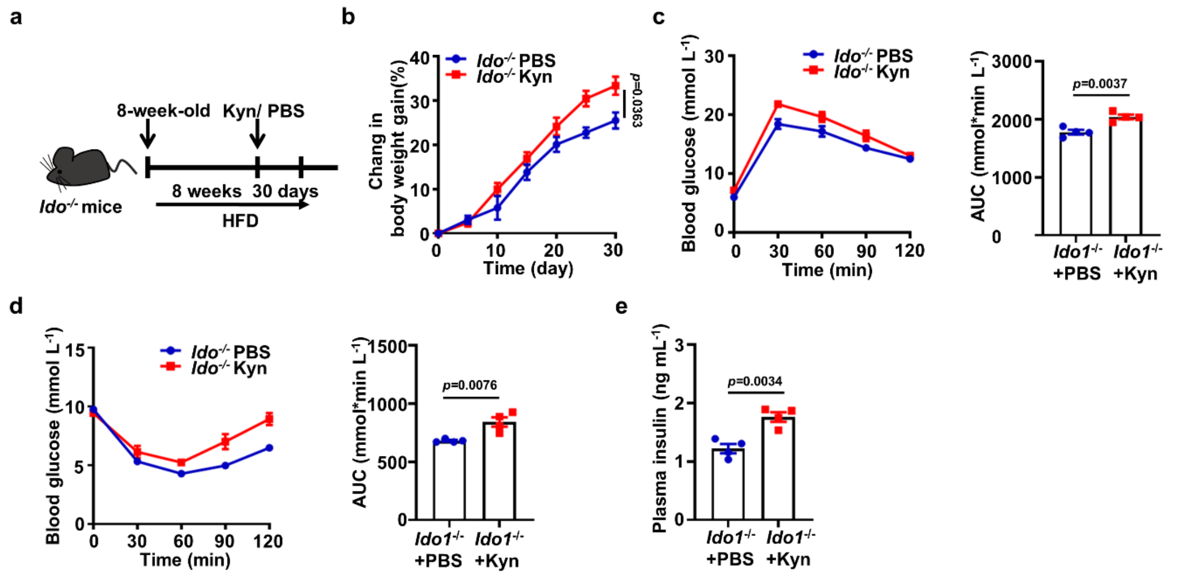

Supplementary Figure 3. Kyn supplementation reverses the phenotype of *Ido*$^{-/-}$ mice in obesity. a, *Ido*$^{-/-}$ mice were fed with HFD for 8 weeks, and then subcutaneously injected with L-Kyn (20 mg kg^{-1} d^{-1}, n = 4) or PBS (n = 4) for another 30 days. **b**, Body weights of Kyn-treated and PBS-treated *Ido*$^{-/-}$ mice. **c**, **d**, GTT (**c**) and ITT (**d**) of Kyn-treated and PBS-treated *Ido*$^{-/-}$ mice (n = 4). **e**, Plasma insulin levels of Kyn-treated and PBS-treated *Ido*$^{-/-}$ mice (n = 4). Data were represented as mean ± SEM. Statistical significance was assessed by two-way ANOVA followed with Bonferroni's multiple comparisons test (**b-d**, left panel) or two-sided Student's *t*-test (**c-d**, right panel**, e**) and significant differences were indicated with *p* values. Source data are provided in the Source Data file.

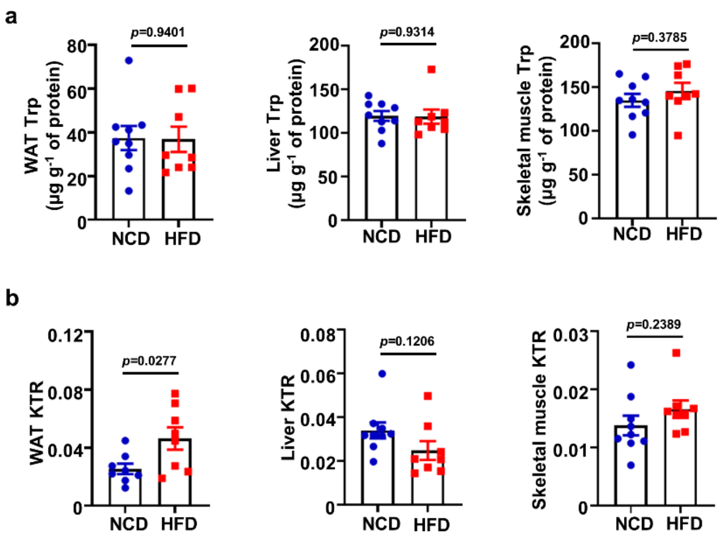

Supplementary Figure 4. Trp level and KTR in WAT, liver and skeletal muscle of NCD and HFD mice. a, Concentration of Trp in WAT, liver and skeletal muscle of NCD and HFD mice (n = 8). **b**, KTR of WAT, liver and skeletal muscle originated from NCD and HFD mice (n = 8). Data were represented as mean ± SEM. Two-sided Student's t-test was used for statistical analysis and significant differences were indicated with p values. Source data are provided in the Source Data file.

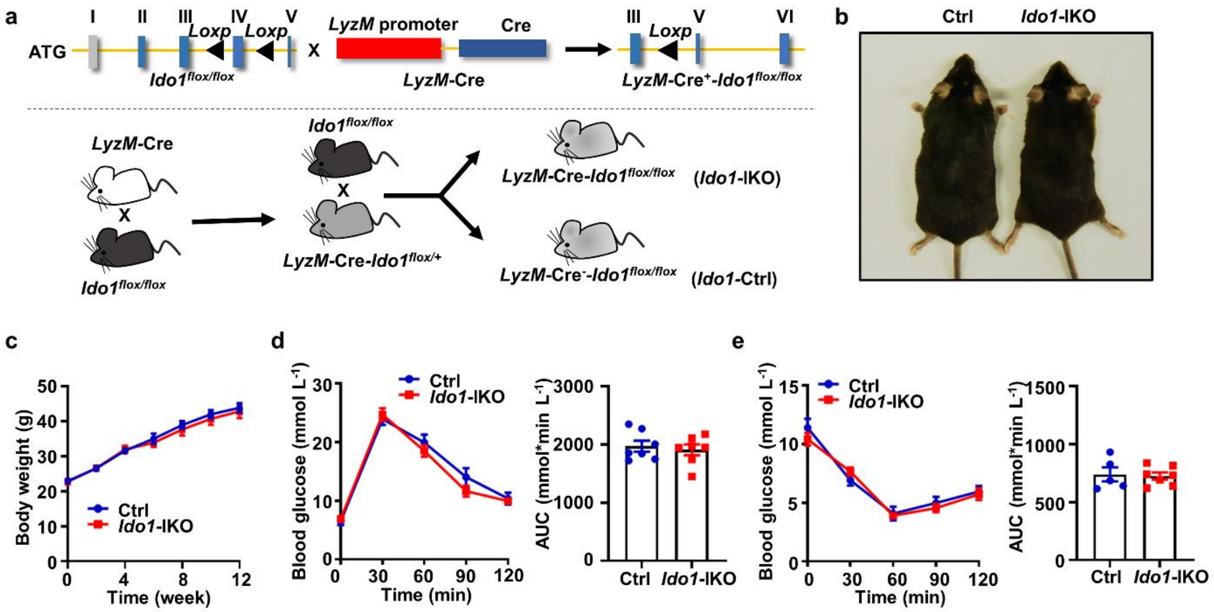

Supplementary Figure 5. Loss of IDO1 in macrophages has no effect on HFD induced obesity and insulin resistance. a, The *Ido1*$^{\text{flox/flox}}$ mice were crossed with the *LyzM*-Cre mice to get the *LyzM*-Cre-*Ido1*$^{\text{flox/flox}}$ mice (*Ido1*-lKO). **b**, Representative images of the Ctrl mice and *Ido1*-lKO mice after 12-week HFD. **c**, Body weight of the Ctrl mice (n = 7) and *Ido1*-lKO mice (n = 7) after 12-week HFD. **d**, **e**, GTT (**d**) and ITT (**e**) in the Ctrl mice (n = 6 for IGTT, n = 5 for ITT) and *Ido1*-lKO mice (n = 8 for IGTT, n = 7 for ITT). Data were represented as mean ± SEM. Statistical significance was assessed by two-way ANOVA followed with Bonferroni's multiple comparisons test (**c-d**). Source data are provided in the Source Data file.

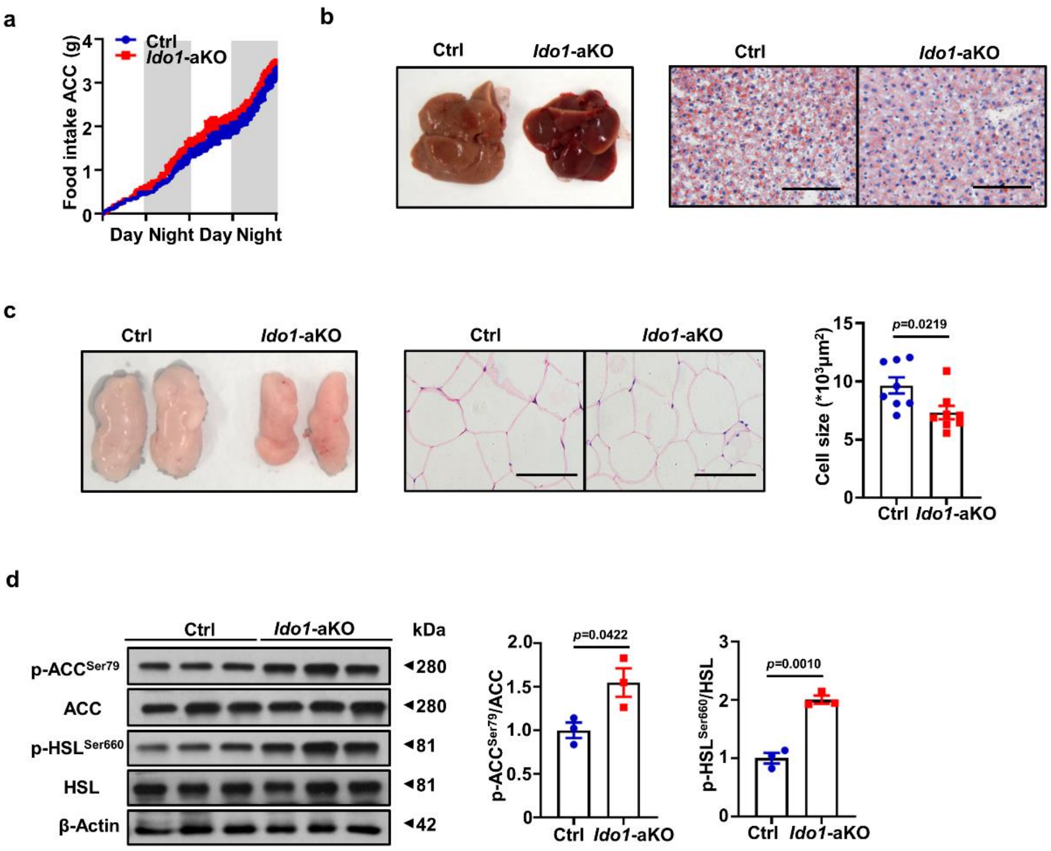

Supplementary Figure 6. *Ido1* deficiency in adipocytes renders the mice with decreased Kyn level and resistance to obesity. a, Food consumptions of the Ctrl and *Ido1*-aKO mice fed with HFD were determined by metabolic cages (n = 4). **b**, Representative images of liver and Oil Red staining of liver (n = 6) in 12-week HFD-fed Ctrl and *Ido1*-aKO mice. The experiments were repeated independently three times. Scale bar, 100 µm. **c**, Representative images of eWAT and H&E staining images of eWAT originated from 12-week HFD-fed Ctrl and *Ido1*-aKO mice (left) and statistics analysis of the adipocyte size (right) (n = 8). Scale bar, 100 µm. **d**, Western blot of p-ACC^{Ser79} /ACC and p-HSL^{Ser660}/HSL in eWAT originated from the Ctrl and *Ido1*-aKO mice with 12-week HFD (n = 3). Data were represented as mean ± SEM. Statistical significance was assessed by two-way ANOVA followed with Bonferroni's multiple comparisons test (**a**) or two-

sided Student's t-test (**c** and **d**) and significant differences were indicated with *p* values. Source data are provided in the Source Data file.

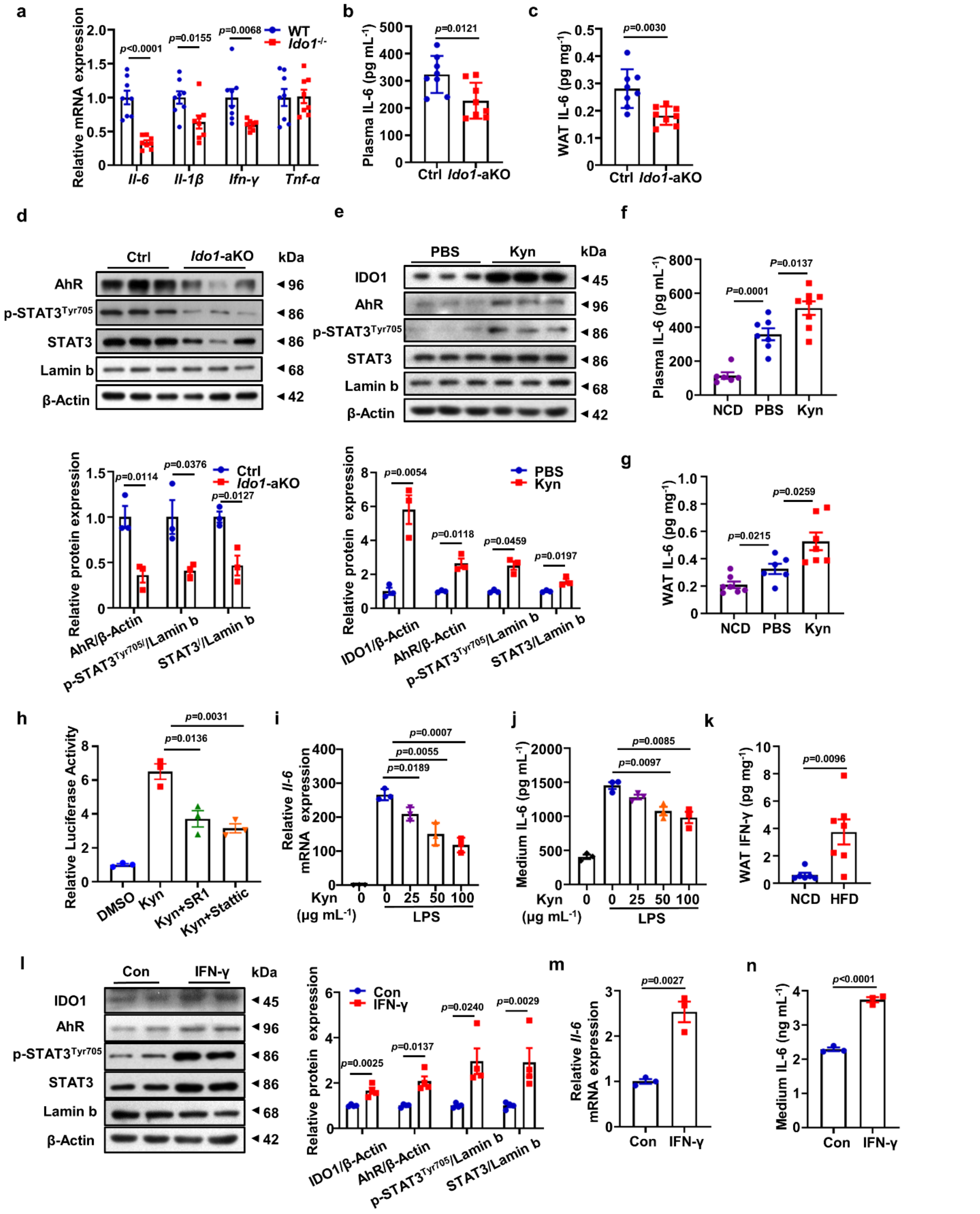

Supplementary Figure 7. a, Transcriptional levels of proinflammatory genes (*Il-6, Il-1β, Ifn-γ,Tnf-α*) in eWAT of HFD-fed Ctrl and *Ido*$^{-/-}$ mice (n = 8). **b, c,** ELISA analysis of IL-6

concentration in plasma (**b**) and eWAT (**c**) from the Ctrl and *Ido1*-aKO mice with 12-week HFD ($n = 8$). **d,** Western blot analysis of AhR, p-STAT3^{Tyr705}/STAT3 of eWAT from the Ctrl and *Ido1*-aKO mice with 12-week HFD ($n = 3$). **e,** IDO1, AhR, p-STAT3^{Tyr705}/STAT3 expression levels of eWAT from PBS-treated and Kyn-treated mice with 12-week HFD were determined by Western blot ($n = 3$). **f,** ELISA analysis of IL-6 concentration in plasma from NCD ($n = 6$), HFD ($n = 7$) and HFD+Kyn ($n = 8$) mice. **g**, ELISA analysis of IL-6 concentration in eWAT from NCD ($n = 7$), HFD ($n = 6$) and HFD+Kyn mice ($n = 7$). **h,** Relative luciferase activity of the cells treated with SR1 or Stattic. **i,** Transcriptional levels of IL-6 in BMDMs treated with 0, 25, 50, 100 μg mL^{-1} Kyn with or without LPS pretreatment ($n = 3$ independent experiments). **j,** ELISA analysis of IL-6 in the culture supernatants of the BMDMs as above ($n = 3$). **k,** ELISA analysis of IFN-γ concentration in eWAT from the NCD ($n = 6$) or HFD ($n = 7$) fed mice. **l**, Western blot results of IDO1, AhR, p-STAT3^{Tyr705}/STAT3 in mature adipocytes stimulated with PBS or IFN-γ (50 ng mL^{-1}) for 3h (n = 4 independent experiments). **m, n,** RT-PCR analysis of *Il-6* expression (**m**) and ELISA analysis of IL-6 concentration in the culture supernatants (**n**) in mature adipocytes stimulated with IFN-γ (50 ng mL^{-1}) for 3h ($n = 3$ independent experiments). Data were represented as mean ± SEM. Statistical significance was assessed by two-sided Student's *t*-test (**a-e**, **k**, **m** and **n**), one-way ANOVA test (**f-h**) or two-way ANOVA test (**i** and **j**) and significant differences were indicated with *p* values. Source data are provided in the Source Data file.

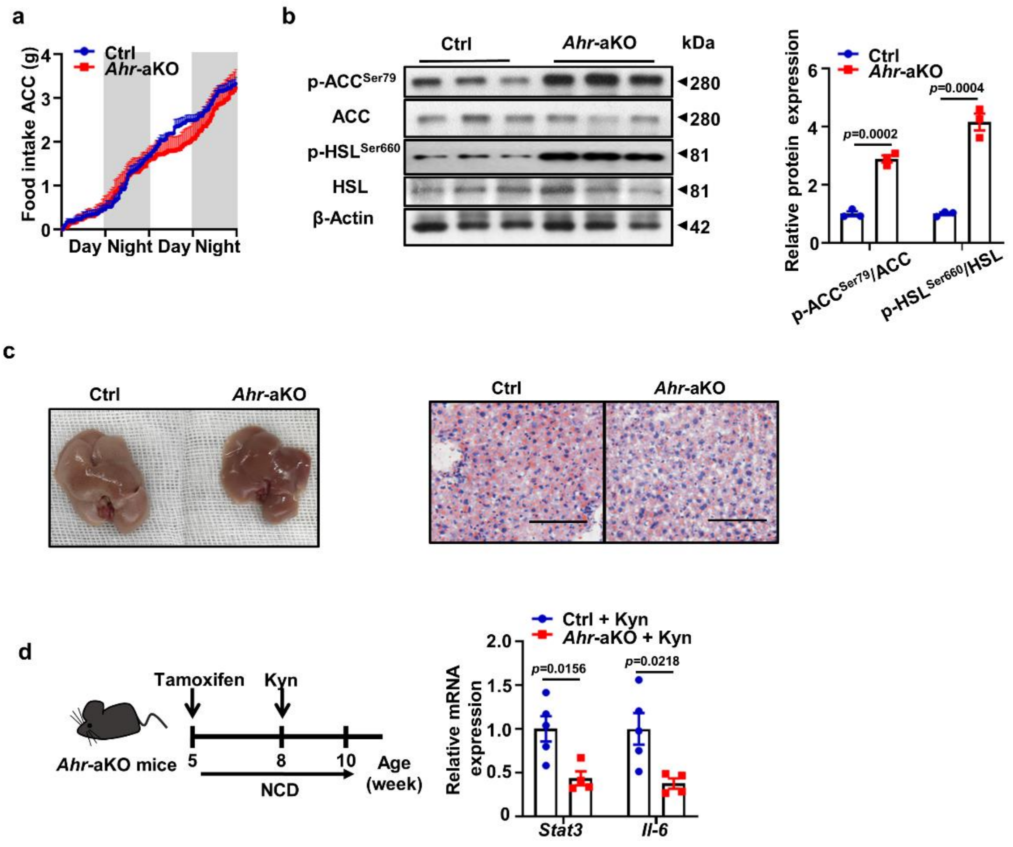

Supplementary Figure 8. Kyn promotes obesity and insulin resistance depending on AhR. a, Cumulative food intake of the Ctrl and *Ahr*-aKO mice with 12-week HFD (n = 4). **b**, Western blot analysis of p-ACC^{Ser79}/ACC, p-HSL^{Ser660}/HSL in eWAT from the Ctrl and *Ahr*-aKO mice. **c**, Representative images of liver and Oil Red staining images of liver (n = 4) originated from the Ctrl and *Ahr*-aKO mice with 12-week HFD. Scale bar, 100 μm. **d**, *Ahr*-aKO mice were randomly divided into two groups, and were respectively injected subcutaneously with L-Kyn (20 mg kg^{-1} d^{-1}, n = 4) or PBS (n = 4) for two weeks. RT-qPCR was used to determine the transcription levels of *Stat3* and *Il-6* in eWAT (n = 4). Data were represented as mean ± SEM. Statistical significance was assessed by two-way ANOVA followed with Bonferroni's multiple comparisons test (**a**) or two-sided Student's t-test (**b** and **d**) and significant differences were indicated with p values. Source data are provided in the Source Data file.

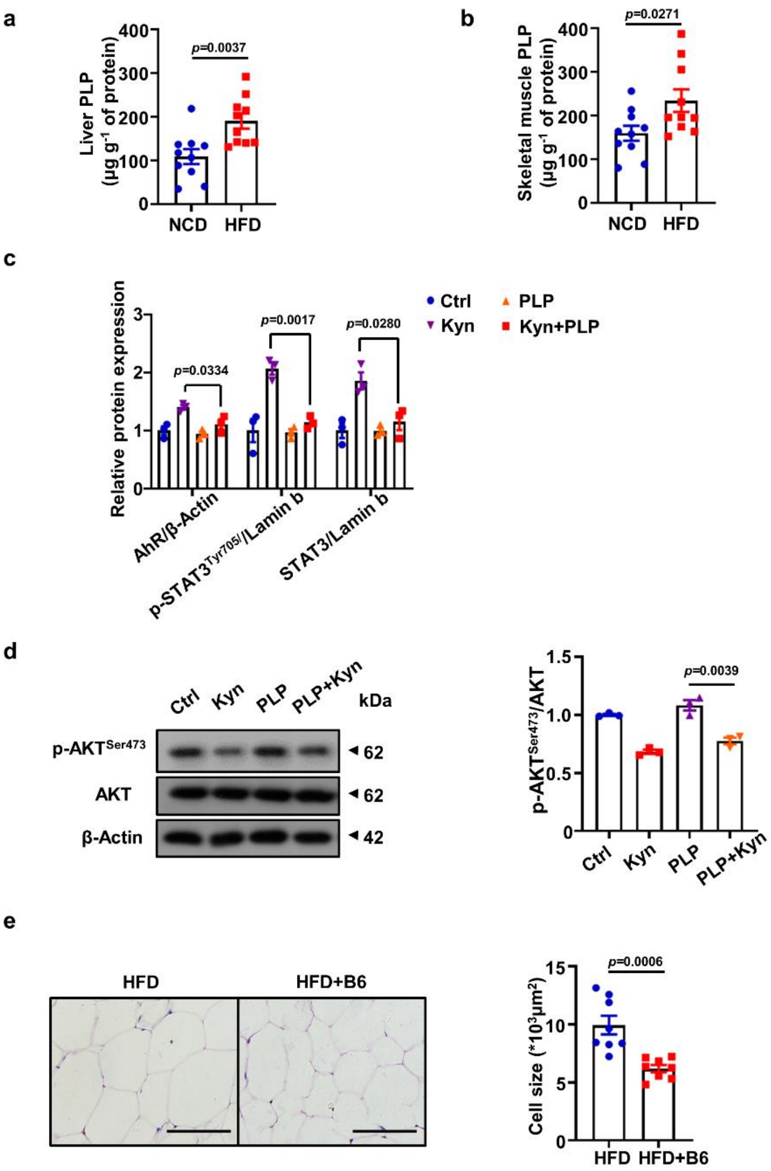

Supplementary Figure 9. Vit-B6 confers protection by facilitating Kyn catabolism. a, **b**, PLP concentration of liver and skeletal muscle originated from the NCD and HFD mice (n = 10). **c**, Quantification of the Western blot bands in Fig. 7d. **d**, Western blot results of p-AKT^{Ser473}/AKT in mature adipocytes with different treatments (n = 3). **e**, Representative H&E staining images of eWAT (left) and the statistics analysis of adipocyte size (right) from HFD-fed WT mice with or without Vit-B6 supplementation (n = 8). Scale bar, 100 μm. Data were represented as mean ± SEM. Two-sided Student's t-test was used for statistical analysis (**a**, **b** and **e**). Statistical significance was

assessed by one-way ANOVA (**c** and **d**) and significant differences were indicated with *p* values.

Source data are provided in the Source Data file.

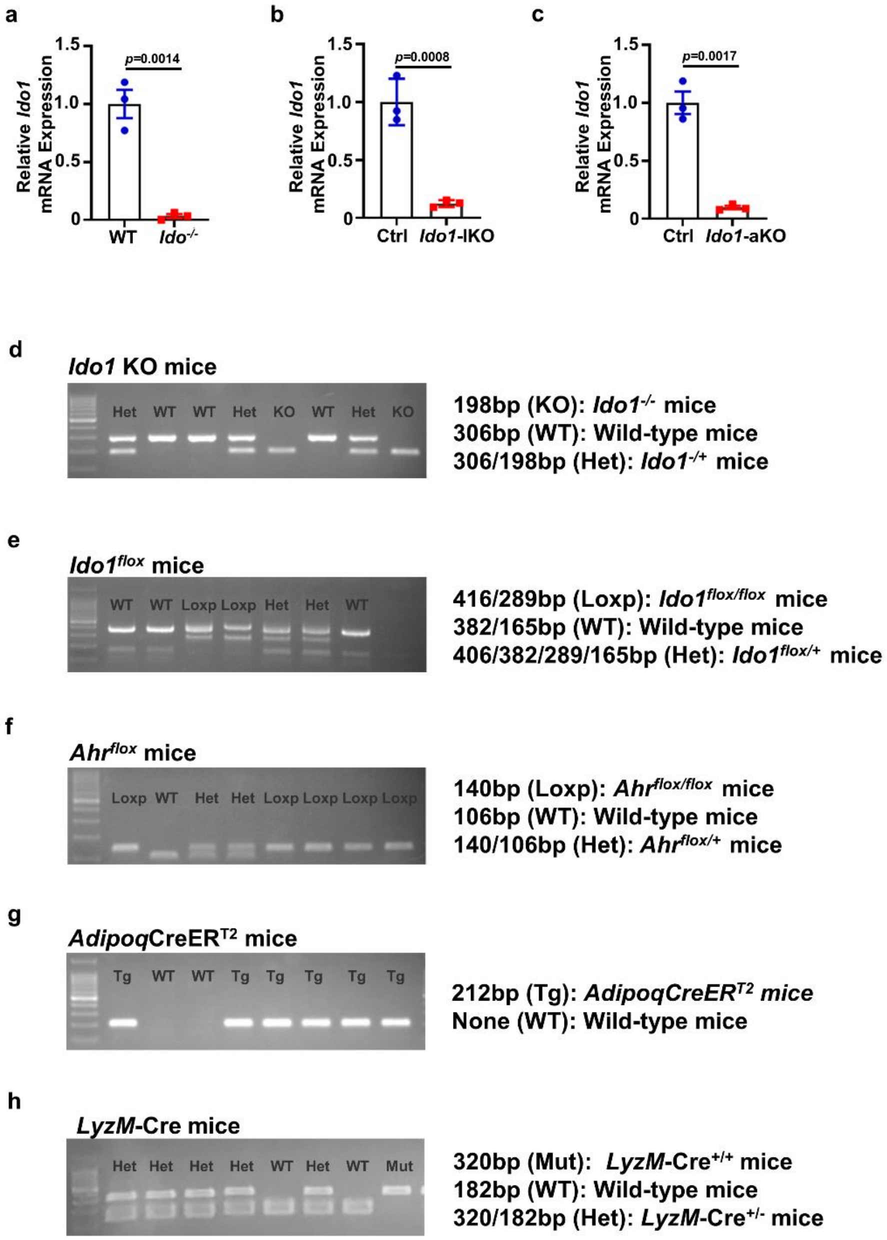

Supplementary Figure 10. Knockout efficiency and genotyping results of mice used in this research. a, Transcriptional levels of *Ido1* in mature adipocytes of *Ido1* KO mice. **b,** Transcriptional levels of *Ido1* in macrophages of *Ido1*-lKO mice**. c,** Transcriptional levels of *Ido1* in mature adipocytes of *Ido1*-aKO mice. **d**, *Ido1* KO mice. **e**, *Ido1*^{flox} mice. **f**, *Ahr*^{flox} mice. **g**, *Adipoq*CreER$^{\text{T2}}$ mice. **h**, *LyzM*-Cre mice. WT, wild-type. Het, heterozygous. Tg, transgenic mice. Mut, mutation. Data were represented as mean ± SEM. Two-sided Student's *t*-test was used for

statistical analysis (**a** - **c**) and significant differences were indicated with *p* values. Source data are provided in the Source Data file.

Supplementary Table 1. Characteristics of subjects from which plasma samples were collected.

Variable	Lean	Overweight	Obese
BMI	18 ≤ BMI < 24	24 ≤ BMI < 28	BMI ≥ 28
Number, *n*	354	268	113
Age, *y*	49.4±0.7	48.2±0.8	46.4±1.4
Female, *%*	68.1	51.9	41.6
BMI, kg/m^2	21.72±0.09	25.65±0.07	30.12±0.18
Waist, *cm*	73.92±0.60	82.43±0.76	93.24±1.14
KTR	0.028±0.0008	0.032±0.0005	0.037±0.0012
Kyn, *ng/mL*	289.12±4.45	336.23±6.00	393.85±12.20
Trp, *mg/mL*	10.58±0.12	10.62±0.13	10.85±0.19
PLP, *ng/Ml*	13.93±0.45	10.52±0.49	7.87±0.42

BMI, body mass index; KTR, Kyn/Trp ratio; Kyn, kynurenine; Trp, tryptophan; PLP, pyridoxal 5'-phosphate.

Supplementary Table 2. Characteristics of subjects from which omental adipose tissue were taken.

Variable	
Age, *y*	41.41 ± 4.04
Female, *n(%)*	9 (40.91%)
BMI, kg/m^2	20.2~48.9
Height, *m*	1.66 ± 0.08
Weight, *kg*	74.98 ± 4.46

Supplementary Table 3. The sequences of the primers that were used in this research.

Genes	Primer
IDO1 (Human)-F	5'-GCCTGATCTCATAGAGTCTGGC-3'
IDO1 (Human)-R	5'-TGCATCCCAGAACTAGACGTGC-3'
ACTB (Human)-F	5'-CACCATTGGCAATGAGCGGTTC-3'
ACTB (Human)-R	5'-AGGTCTTTGCGGATGTCCACGT-3'
Ido1 (Mouse)-F	5'-GGATGCGTGACTTTGTGGACC-3'
Ido1 (Mouse)-R	5'-TCAAGACTCTGGAAGATGCTGCTC-3'
Ido1 -(KO) (Mouse)-F	5'-TGGGCCTGCCTCCTATTCT-3'
Ido1-(KO) (Mouse)-R	5'-AGAAGCTGCGATTTCCACCA-3'
Actin (Mouse)-F	5'-TCAAGACTCTGGAAGATGCTGCTC-3'
Actin (Mouse)-R	5'-CTCCAGCTGTGGTGGTGAA-3'
C/ebpα (Mouse)-F	5'-CAAGAACAGCAACGAGTACCG-3'
C/ebpα (Mouse)-R	5'-GTCACTGGTCAACTCCAGCAC-3'
Pparγ (Mouse)-F	5'-TCGCTGATGCACTGCCTATG-3'
Pparγ (Mouse)-R	5'-GAGAGGTCCACAGAGCTGATT-3'
Fabp4 (Mouse)-F	5'-AAGGTGAAGAGCATCATAACCCT-3'
Fabp4 (Mouse)-R	5'-TCACGCCTTTCATAACACATTCC-3'
Il-6 (Mouse)-F	5'-ATGGATGCTACCAAACTGGAT-3'
Il-6 (Mouse)-R	5'-TGAAGGACTCTGGCTTTGTCT-3'
Stat3 (Mouse)-F	CAATACCATTGACCTGCCGAT
Stat3 (Mouse)-R	GAGCGACTCAAACTGCCCT
