# Supplementary material for: Adipocyte-derived kynurenine promotes obesity and insulin resistance by activating the AhR/STAT3/IL-6 signaling
Source: Nat Commun. 2022 Jun 17;13:3489. doi: 10.1038/s41467-022-31126-5 (PMC9205899; doi:10.1038/s41467-022-31126-5)
Supplement: Supplementary file 3 — Reporting Summary [file 41467_2022_31126_MOESM3_ESM.pdf]

nature portfolio

Corresponding author(s): Fei Xiong, Qing Zhou and Cong-Yi Wang

Last updated by author(s): May 23, 2022

Reporting Summary

Nature Portfolio wishes to improve the reproducibility of the work that we publish. This form provides structure for consistency and transparency in reporting. For further information on Nature Portfolio policies, see our Editorial Policies and the Editorial Policy Checklist.

Statistics

For all statistical analyses, confirm that the following items are present in the figure legend, table legend, main text, or Methods section.

n/a	Confirmed	
☐	☒	The exact sample size (*n*) for each experimental group/condition, given as a discrete number and unit of measurement
☐	☒	A statement on whether measurements were taken from distinct samples or whether the same sample was measured repeatedly
☐	☒	The statistical test(s) used AND whether they are one- or two-sided<br>*Only common tests should be described solely by name; describe more complex techniques in the Methods section.*
☒	☐	A description of all covariates tested
☐	☒	A description of any assumptions or corrections, such as tests of normality and adjustment for multiple comparisons
☐	☒	A full description of the statistical parameters including central tendency (e.g. means) or other basic estimates (e.g. regression coefficient) AND variation (e.g. standard deviation) or associated estimates of uncertainty (e.g. confidence intervals)
☐	☒	For null hypothesis testing, the test statistic (e.g. *F*, *t*, *r*) with confidence intervals, effect sizes, degrees of freedom and *P* value noted<br>*Give P values as exact values whenever suitable.*
☒	☐	For Bayesian analysis, information on the choice of priors and Markov chain Monte Carlo settings
☒	☐	For hierarchical and complex designs, identification of the appropriate level for tests and full reporting of outcomes
☐	☒	Estimates of effect sizes (e.g. Cohen's *d*, Pearson's *r*), indicating how they were calculated

Our web collection on statistics for biologists contains articles on many of the points above.

Software and code

Policy information about availability of computer code

Data collection	HPLC system (Shimsdzu, Kyoto, Japan) equipped with Ultimate XB-C18 HPLC column was used for metabolite data.<br>OLYMPUS Upright microscope BX53 was used to acquire microscopy images.<br>MACSQuantTM was applied to acquire flow cytometry data.<br>Columbus Instruments metabolic cages were used for collected metabolic studies.
Data analysis	Image J 1.46r software was used to quantify adipose size. GraphPad Prism version 5.0 software was applied for data analysis. FlowJo software v10.5.3 was used to analyze flow cytometry data. Gelpro 32 software was used to analyze western blot data. Data were represented as mean ± SEM. The normality of the data was tested using the Shapiro-Wilk normality test. Data were compared using unpaired, two-tailed Student's t-test, one-way ANOVA with the Holm-Sidak multiple-comparisons test with corrections, Spearman's correlation or two-way ANOVA followed with Bonferroni's multiple comparisons test when applicable. Statistical analyses for correlation were performed using Spearman's correlation. In all cases, $p < 0.05$ was considered as statistical significance. All statistical tests were performed by the GraphPad Prism version 5.0 software (La Jolla, CA, USA).The images were created by using PowerPoint 2016 and CorelDRAW X6 software.

For manuscripts utilizing custom algorithms or software that are central to the research but not yet described in published literature, software must be made available to editors and reviewers. We strongly encourage code deposition in a community repository (e.g. GitHub). See the Nature Portfolio guidelines for submitting code & software for further information.

Data

Policy information about availability of data

All manuscripts must include a data availability statement. This statement should provide the following information, where applicable:

- Accession codes, unique identifiers, or web links for publicly available datasets
- A description of any restrictions on data availability
- For clinical datasets or third party data, please ensure that the statement adheres to our policy

The authors declare that the main data supporting the findings of this study are available within the article and its Supplementary Information. The source data are provided with the paper as a Source Data file.

Field-specific reporting

Please select the one below that is the best fit for your research. If you are not sure, read the appropriate sections before making your selection.

☒ Life sciences ☐ Behavioural & social sciences ☐ Ecological, evolutionary & environmental sciences

For a reference copy of the document with all sections, see nature.com/documents/nr-reporting-summary-flat.pdf

Life sciences study design

All studies must disclose on these points even when the disclosure is negative.

Sample size	The sample size was chosen to assure significant statistical differences and reproducibility of the results. The maximum number of available mice for each experiment was used respecting the guidelines of animal welfare. For in vivo experiments, cohort size was determined by types of experiment and availability of animals as littermates were used in each experiment and our sample size ranging from 4-10 mice per group. For in vitro experiments, we repeated at least 3 times in each group.No statistical method was used to pre-select the sample size. The sample size was determined based on similar previous studies of our laboratory and on previous experiments using similar methodologies. Detailed sample size were described in the figure legends.
Data exclusions	No data were excluded.
Replication	All data are the result of independently-repeated experiments with independent biological sample. Experiments were repeated independently at least twice with similar results in repeated experiments.
Randomization	Mice were randomized into the different groups. Cells and tissues from human donors were also randomized.
Blinding	The investigators were blinded to group allocation during data collection and analysis.

Reporting for specific materials, systems and methods

We require information from authors about some types of materials, experimental systems and methods used in many studies. Here, indicate whether each material, system or method listed is relevant to your study. If you are not sure if a list item applies to your research, read the appropriate section before selecting a response.

Materials & experimental systems

n/a	Involved in the study	
☐	☒	Antibodies
☐	☒	Eukaryotic cell lines
☒	☐	Palaeontology and archaeology
☐	☒	Animals and other organisms
☐	☒	Human research participants
☒	☐	Clinical data
☒	☐	Dual use research of concern

Methods

n/a	Involved in the study	
☒	☐	ChIP-seq
☐	☒	Flow cytometry
☒	☐	MRI-based neuroimaging

Antibodies

Antibodies used	rabbit anti-phospho-Akt (Ser473) (Cell Signaling Technology, 4060s, MA, USA, 1: 1000)<br>rabbit anti-AKT (Cell Signaling Technology, 4685s, MA, USA, 1: 1000)<br>rabbit anti-phopho-HSL (Ser660) Antibody (Cell Signaling Technology, 4126s, MA, USA, 1: 1000)<br>rabbit anti-phospho-Acetyl-CoA Carboxylase (Ser79) Antibody (Cell Signaling Technology, 3661s, MA, USA, 1: 1000)<br>rabbit anti-ACC (Cell Signaling Technology, 3676s, MA, USA, 1: 1000)

rabbit anti-phospho-GSK-3β (Ser9) (Cell Signaling Technology, 9323T, MA, USA, 1: 1000)
rabbit anti-GSK3β (Cell Signaling Technology, 12456s, MA, USA, 1: 1000)
rabbit anti-phospho-STAT3(Tyr705) (Cell Signaling Technology, 9145s, MA, USA, 1: 1000)
rabbit anti-STAT3 (Cell Signaling Technology, 4904s, MA, USA, 1: 1000)
mouse anti-Ah Receptor (A-2) (Santa Cruz Biotechnology, Sc-398877,CA, USA, 1:1000)
Polyclonal goat anti-Ah Receptor(N-19) (Santa Cruz Biotechnology, Sc-8088X,CA, USA, 1:100)
Polyclonal rabbit anti-Lamin B1 (Santa Cruz Biotechnology, Sc-20682, CA, USA, 1:1000)
Polyclonal rabbit anti-β-Actin (Santa Cruz Biotechnology, Sc-47778, CA, USA, 1:1000)
Polyclonal rabbit anti-PEPCK (Santa Cruz Biotechnology, Sc-32879, CA, USA, 1:1000)
rabbit anti-G6pase (Santa Cruz Biotechnology, Sc-25840, CA, USA, 1:1000)
Polyclonal rabbit anti-HSL (Santa Cruz Biotechnology, Sc-25843, CA, USA, 1:1000)
mouse anti-IDO1 (Biolegend, 122402, San Diego, CA, USA, 1:1000)
PE anti-mouse F4/80 (Biolegend, 123110, San Diego, CA, USA, 1:400)
FITC anti-mouse CD11b (Biolegend, 101206, San Diego, CA, USA, 1:400)
APC anti-mouse CD11c (Biolegend, 117310, San Diego, CA, USA, 1:400)
PE/Cy7 anti-mouse CD206 (Biolegend, 141720, San Diego, CA, USA, 1:400)

Validation

rabbit anti-phospho-AKT(Ser473) https://www.cellsignal.cn/products/primary-antibodies/phospho-akt-ser473-d9e-xp-rabbit-mab/4060
rabbit anti-AKT https://www.cellsignal.cn/products/primary-antibodies/akt-pan-11e7-rabbit-mab/4685?site-search-type=Products&N=4294956287&Ntt=4685&fromPage=plp&_requestid=39945
rabbit anti-phospho-HSL (Ser660) Antibody https://www.cellsignal.cn/products/primary-antibodies/phospho-hsl-ser660-antibody/4126?site-search-type=Products&N=4294956287&Ntt=4126&fromPage=plp&_requestid=42406
rabbit anti-phospho-Acetyl-CoA Carboxylase (Ser79) Antibody https://www.cellsignal.cn/products/primary-antibodies/phospho-acetyl-coa-carboxylase-ser79-antibody/3661?site-search-type=Products&N=4294956287&Ntt=3661&fromPage=plp&_requestid=42556
rabbit anti-Acetyl-CoA Carboxylase https://www.cellsignal.cn/products/primary-antibodies/acetyl-coa-carboxylase-c83b10-rabbit-mab/3676?site-search-type=Products&N=4294956287&Ntt=3676&fromPage=plp&_requestid=43258
rabbit anti-phospho-GSK-3β (Ser9) https://www.cellsignal.cn/products/primary-antibodies/phospho-gsk-3b-ser9-5b3-rabbit-mab/9323?site-search-type=Products&N=4294956287&Ntt=9323&fromPage=plp&_requestid=43522
rabbit anti-GSK3β https://www.cellsignal.cn/products/primary-antibodies/gsk-3b-d5c5z-xp-rabbit-mab/12456?site-search-type=Products&N=4294956287&Ntt=12456&fromPage=plp&_requestid=43818
rabbit anti-phospho-STAT3(Tyr705) https://www.cellsignal.cn/products/primary-antibodies/phospho-stat3-tyr705-d3a7-xp-rabbit-mab/9145?site-search-type=Products&N=4294956287&Ntt=9145&fromPage=plp&_requestid=43970
rabbit anti-STAT3 https://www.cellsignal.cn/products/primary-antibodies/stat3-79d7-rabbit-mab/4904?site-search-type=Products&N=4294956287&Ntt=4904&fromPage=plp&_requestid=44355
mouse anti-Ah Receptor (A-2) https://www.scbt.com/p/ah-receptor-antibody-a-2?requestFrom=search
Polyclonal goat anti-Ah Receptor(N-19) https://www.scbt.com/zh/p/ah-receptor-antibody-n-19?requestFrom=search
Polyclonal rabbit anti-Lamin B1 https://www.scbt.com/p/lamin-b1-antibody-h-90?requestFrom=search
Polyclonal rabbit anti-β-Actin https://www.scbt.com/p/beta-actin-antibody-c4?requestFrom=search
Polyclonal rabbit anti-PEPCK (H-300) https://www.scbt.com/p/pepck-antibody-h-300?requestFrom=search
rabbit anti-G6pase (H-60) https://www.scbt.com/zh/p/g6pase-alpha-antibody-h-60
Polyclonal rabbit anti-HSL (H-300) https://www.scbt.com/zh/p/hsl-antibody-h-300?requestFrom=search
mouse anti-IDO1 https://www.biolegend.com/en-us/products/purified-anti-ido1-antibody-8343
PE anti-mouse F4/80 https://www.biolegend.com/en-us/products/pe-anti-mouse-f4-80-antibody-4068
FITC anti-mouse CD11b https://www.biolegend.com/en-us/products/fitc-anti-mouse-human-cd11b-antibody-347
APC anti-mouse CD11c https://www.biolegend.com/en-us/products/apc-anti-mouse-cd11c-antibody-1813
PE/Cy7 anti-mouse CD206 https://www.biolegend.com/en-us/products/pe-cyanine7-anti-mouse-cd206-mmr-antibody-8631

Eukaryotic cell lines

Policy information about cell lines

Cell line source(s)	3T3-LI cells (ATCC CL-173) were purchased from ATCC
Authentication	The cell line was not authenticated
Mycoplasma contamination	Cells tested negative for mycoplasm contamination
Commonly misidentified lines (See ICLAC register)	No commonly misidentified cell lines were used in the study

Animals and other organisms

Policy information about studies involving animals; ARRIVE guidelines recommended for reporting animal research

Laboratory animals	Male mice (C57BL/6 background, 8-10 weeks) were used in this study. Housing conditions:Temperature:20-24°C; Humidity:45-65%, 12/12 hours light/dark cycle.
Wild animals	No wild animals were used in the study.
Field-collected samples	The study did not involve samples collected from the field.

Ethics oversight | All animal procedures were approved by the Tongji Hospital Animal Care and Use Committee in line with the USA National Institutes of Health (NIH) guidelines.

Note that full information on the approval of the study protocol must also be provided in the manuscript.

Human research participants

Policy information about studies involving human research participants

Population characteristics	Fasting plasma samples were collected in Tongji Hospital from year 2016 to 2017. Subjects with renal or liver dysfunction were excluded from our study. Clinical characteristics of participants are summarized in Supplementary Table 1. Omental adipose tissue was obtained from patients (n = 22) undergoing abdominal surgery for benign diseases. Clinical characteristics of participants are summarized inSupplementary Table 2. All patients were devoid of any evident systemic disease, any chronic infection or previous myocardial infarction.
Recruitment	Fasting plasma samples were collected in Tongji Hospital from year 2016 to 2017. Omental adipose tissue was obtained from patients (n = 22) undergoing abdominal surgery for benign diseases. Participants were recruited randomly and without self-selection.
Ethics oversight	Human studies were conducted in accordance with the NIH guidelines and were approved by the Institutional Review Board (IRB) of Tongji Hospital (TJ-IRB20160601, TJ-IRB20160602).

Note that full information on the approval of the study protocol must also be provided in the manuscript.

Flow Cytometry

Plots

Confirm that:

☒ The axis labels state the marker and fluorochrome used (e.g. CD4-FITC).

☒ The axis scales are clearly visible. Include numbers along axes only for bottom left plot of group (a 'group' is an analysis of identical markers).

☒ All plots are contour plots with outliers or pseudocolor plots.

☒ A numerical value for number of cells or percentage (with statistics) is provided.

Methodology

Sample preparation	Fresh adipose tissue was cut into small pieces and transferred to a 50 mL centrifuge tube. Then, an equal volume of digestion medium (2 mg mL-1 Collagenase Type I containing 0.5 mg mL-1 CaCl2) was added into the adipose tissue and mixed thoroughly. The mixture was digested at 37 °C with constant agitation at 150 rpm for 20-30 minutes until the cells were completely homogenous. Digestion was stopped by adding an equal volume of complete medium (DMEM, containing 10% FBS). After centrifuged at 300 g for 5 minutes, SVFs were in the bottom and mature adipocytes layered on the top. The oil was abandoned and the mature adipocyte layer was moved to a new 15 mL tube. After centrifuged at 100 g for 2-3 times, the oil layer was removed and mature adipocytes were obtained. For SVFs, the cell pellet was resuspended in 5 mL medium, and then filtered with a 70 μm cell strainer. The cell suspension was transferred to a 15 mL tube and centrifuged at 300 g for 5 min. Then, SVFs were in the bottom of the tube. For surface markers, the cells were stained in PBS containing 1% BSA with indicated antibodies for 30min on ice. For intracellular markers, the cells were first fixed with Fixation Buffer (420801; Biolegend, San Diego, CA, USA) at 4°C for 30min and then stained in Permeabilization Wash Buffer (421002; Biolegend, San Diego, CA, USA) with relevant antibodies at 4°C for 30min.
Instrument	MACSQuantTM (Miltenyi Biotec, Auburn, CA, USA)
Software	FlowJo software v10.5.3
Cell population abundance	No cell sorting was performed.
Gating strategy	Cells were gated by FSC/SSC gates and then FSC/FSC-width to select single cells. After that the detail gating strategy was showed as follows:<br>Mouse macrophages: F4/80+,CD11b+<br>Mouse M1 macrophages: F4/80+,CD11b+, CD11c+, CD206-<br>Mouse M2 macrophages: F4/80+,CD11b+, CD11c-, CD206+

☐ Tick this box to confirm that a figure exemplifying the gating strategy is provided in the Supplementary Information.
